# Characteristics and prognostic factors of bacterial meningitis in the intensive care unit: a prospective nationwide cohort study

**DOI:** 10.1186/s13613-023-01218-6

**Published:** 2023-12-06

**Authors:** Nora Chekrouni, Merel Kroon, Evelien H. G. M. Drost, Thijs M. van Soest, Merijn. W. Bijlsma, Matthijs C. Brouwer, Diederik van de Beek

**Affiliations:** Amsterdam UMC, Department of Neurology, University of Amsterdam, Amsterdam Neuroscience, Meibergdreef, PO Box 22660, 1100DD Amsterdam, The Netherlands

**Keywords:** Bacterial meningitis, Intensive care unit, Admission, Prognostic factors, Unfavourable outcome

## Abstract

**Background:**

Patients with bacterial meningitis can be severely ill necessitating intensive care unit (ICU) treatment. Here, we describe clinical features and prognostic factors of adults with bacterial meningitis admitted to the ICU in a nationwide prospective cohort study.

**Methods:**

We prospectively assessed clinical features and outcome of adults (age > 16 years) with community-acquired bacterial meningitis included in the MeninGene study between March 1, 2006 and July 1, 2022, that were initially admitted to the ICU. We identified independent predictors for initial ICU admission and for unfavourable outcome (Glasgow Outcome Scale score between 1–4) by multivariable logistic regression.

**Results:**

A total of 2709 episodes of bacterial meningitis were included, of which 1369 (51%) were initially admitted to the ICU. We observed a decrease in proportion of patients being admitted to the ICU during the Covid-19 pandemic in 2020 (decreased to 39%, *p* = 0.004). Median age of the 1369 patients initially admitted to the ICU was 61 years (IQR 49–69), and the rates of unfavourable outcome (47%) and mortality (22%) were high. During the Covid-19 pandemic, we observed a trend towards an increase in unfavourable outcome. Prognostic factors predictive for initial ICU admission were younger age, immunocompromised state, male sex, factors associated with pneumococcal meningitis, and those indicative of systemic compromise. Independent predictors for unfavourable outcome in the initial ICU cohort were advanced age, admittance to an academic hospital, cranial nerve palsies or seizures on admission, low leukocyte count in blood, high C-reactive protein in blood, low CSF: blood glucose ratio, listerial meningitis, need for mechanical ventilation, circulatory shock and persistent fever. 204 of 1340 episodes (15%) that were initially not admitted to the ICU were secondarily transferred to the ICU. The rates of unfavourable outcome (66%) and mortality (30%) in this group were high.

**Conclusions:**

The majority of patients with community-acquired bacterial meningitis are admitted to the ICU, and the unfavourable outcome and mortality rates of these patients remain high. Patients that are initially admitted to non-ICU wards but secondarily transferred to the ICU also had very high rates of unfavourable outcome.

**Supplementary Information:**

The online version contains supplementary material available at 10.1186/s13613-023-01218-6.

## Take home message

**Table:** 

This study shows that the majority of patients with bacterial meningitis need intensive care unit (ICU) monitoring and/ or treatment. The rate of unfavourable outcome and mortality of patients initially admitted to the ICU remains high. Patients transferred to the ICU later during admission have a poor outcome which raises the question whether a more liberal ICU admission policy on initial presentation could have improved outcome, as early recognition and treatment of circulatory shock and respiratory failure is crucial for effective treatment of these complications.	

## Introduction

The case-fatality rate of bacterial meningitis is high and depends on the causative pathogen, ranging from 5% for *Neisseria meningitidis* and up to 30% for *Streptococcus pneumoniae* [1–3]. About half of the survivors of this disease suffer from neurological sequelae, such as focal cerebral deficits, hearing impairment, and cognitive impairment [1–5]. Although the implementation of conjugate vaccines against the most common causative pathogens has decreased the incidence of the disease [6–8], the worldwide disease burden is high [5, 9].

Cohort studies have identified several factors prognostic for unfavourable outcome in adults with bacterial meningitis: older age, signs indicative of systemic compromise—such as low blood pressure, high heart rate, high serum inflammatory markers—a low level of consciousness, factors indicative of *S. pneumoniae* infection—such as otitis or pneumonia—delayed antimicrobial treatment, no adjunctive dexamethasone treatment, infection with an antimicrobial resistant pathogen, and host genetic factors [1, 2, 7, 8, 10–15]. Identification of prognostic factors associated with systemic compromise and low level of consciousness in bacterial meningitis has led to the recommendation for an aggressive supportive approach requiring intensive care unit (ICU) admission [5, 16, 17]. However, studies evaluating patients with community-acquired bacterial meningitis on the ICU are mostly retrospective or focussed on specific pathogens only [18–21].

In 2006, we started a prospective cohort study on community-acquired bacterial meningitis (MeninGene) in adults in the Netherlands [2, 8, 15]. Here, we report data of this cohort focussing on patients with community-acquired bacterial meningitis admitted to the ICU, either initially (from the emergency department) or during clinical course (transfer from a non-ICU ward). The main objective was to assess clinical features and to identify independent predictors for initial ICU admission and for unfavourable outcome. The secondary objective was to study the proportion of bacterial meningitis patients initially admitted to the ICU over time.

## Methods

### Patients

The MeninGene study is an ongoing nationwide, prospective cohort study conducted in the Netherlands, with the goal to identify host and pathogen risk factors that can influence susceptibility and outcome of bacterial meningitis [1, 2]. The study started in 2006 and patients aged 17 years or older diagnosed with community-acquired bacterial meningitis are included. The MeninGene investigators are notified by either the treating physician or the Netherlands Reference Laboratory for Bacterial Meningitis (NRLBM) on possible cases of bacterial meningitis. The NRLBM receives approximately 85% of all positive cerebrospinal fluid and blood samples of patients with bacterial meningitis in the Netherlands. The patients, or their legal representatives, are then contacted to receive written information on the study protocol and obtain informed consent for inclusion.

### Inclusion and exclusion criteria

Patients are eligible for inclusion in the MeninGene study if they have a positive cerebrospinal fluid (CSF) culture, or at least one of the following findings in CSF predictive for bacterial meningitis (according to the Spanos criteria): glucose < 1.9 mmol/L, CSF serum glucose ratio < 0.23, protein concentration > 2.20 g/L, white cell count > 2000 cells/mm^3^ or CSF neutrophil count > 1180 cells/mm^3^ [22] in combination with a positive CSF PCR, CSF antigen or blood culture. Patients with a neurosurgical operation in the previous month, head trauma in the previous month, neurosurgical devices in situ, or a hospital-acquired bacterial meningitis (HABM) developed during admission or within 1 week after discharge were excluded. 

### Data collection and definitions

Data are collected prospectively using an online Case Record Form (eCRF). Baseline characteristics, symptoms, results of neurological examinations, clinical course, treatment and outcome are registered. Also hospital type (e.g. academic, top clinical or regional) is registered. Predisposing factors for developing bacterial meningitis were defined as an extra meningeal infection focus (otitis, sinusitis, endocarditis or pneumonia), immunocompromised state (defined as having active cancer, medication treated diabetes, HIV, asplenia, alcoholism or receiving immunosuppressive treatment), or having a CSF leak or a recurrent meningitis episode. Altered mental status was defined as a Glasgow Coma Scale score (GCS) of < 14, and coma as a GCS ≤ 8. Admission wards were divided into ICU and non-ICU wards (medium care, neurology ward or others). Persistent fever was defined as fever lasting for longer than 24 h after administration of antibiotics. Outcome upon hospital discharge was assessed using the Glasgow Outcome Score (GOS), which is a widely used and validated outcome scale ranging from 1 (death) to 5 (mild or no disability) [23]. Unfavourable outcome was defined as GOS score of 1–4, and a favourable outcome as a GOS score of 5.

### Statistical analysis

Categorical variables were expressed using proportions and counts, and continuous variables using the median with interquartile range (IQR). The proportion of episodes admitted to the ICU over time was calculated per epidemiological year (July 1 – June 30). Wald confidence intervals for proportions were estimated using binomial distribution. Comparisons were made using the Mann–Whitney U test for continuous variables and Fisher’s exact test for categorical variables. Logistic regression was used to analyse the association between predictor factors and either initial ICU admission or unfavourable outcome. Possible prognostic factors were selected based on clinical experience and previous studies [18, 20]. The assumption of linearity between a continuous variable and the (log odds of the) outcome was assessed with the Hosmer–Lemeshow goodness-of-fit test and visual inspection. In the absence of a linear relationship, the continuous variable was categorized. Missing data (4.7% of total values) were imputed using multiple imputation, by combining 5 imputed datasets based on all available prognostic factors [24]. All tests were 2-tailed and statistical significance was defined as p < 0.05. All statistical analyses were performed using IBM SPSS Statistics for Windows (v.28) and R studio (V4.0.3).

## Results

**Fig. 1:**
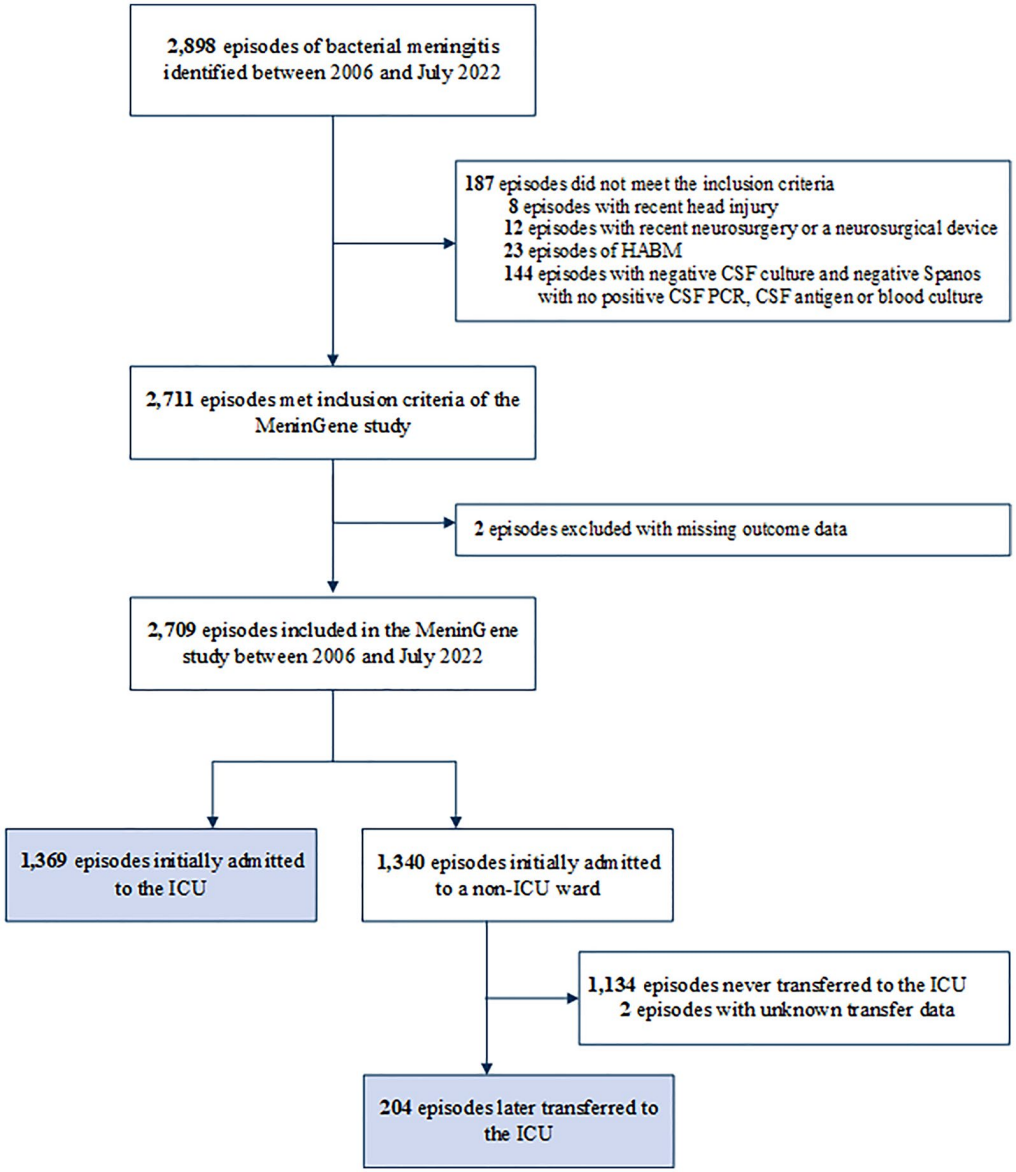
Flowchart with selection of inclusions. *ICU* intensive care unit, *HABM* hospital-acquired bacterial meningitis, *CSF* cerebrospinal fluid, *PCR* polymerase chain reaction

Between March 1, 2006 and July 1, 2022, 2709 episodes of community-acquired bacterial meningitis, occurring in 2654 patients were included in the MeninGene study; 1369 of these 2709 episodes (51%) were initially admitted to the ICU (Fig. 1). Of the episodes initially admitted to a non-ICU ward (*N* = 1340), 204 episodes (15%) were transferred to the ICU during clinical course. The proportion of episodes initially admitted to the ICU was relatively stable over the period 2006–2019, ranging between 46 and 56% (Additional file 1: Figure S1). However, in 2020 there was a sudden decrease to 35% (32 of 91 episodes; *p* = 0.004), after which the proportion increased again to 50% (78 of 155 episodes) in 2021. The baseline characteristics of the 2020 cohort did not differ compared to previous years (Additional file 2: Table S1).

The median age of the 1369 episodes initially admitted to the ICU was 61 years (IQR 49–69) and 632 of 1369 (46%) episodes occurred in females (Table 1). An extra meningeal infection focus was identified in 656 of 1356 (48%) episodes, and 458 of 1369 episodes (33%) were immunocompromised. The triad of neck stiffness (866 of 1216 [71%]), fever (979 of 1325 [74%]) and a decreased level of consciousness (Glasgow Coma Scale score < 14, 1155 of 1360 [85%]) was present in 585 of 1254 episodes (47%). The median Glasgow Coma Scale score (GCS) was 10 (IQR 8–12) and 431 of 1360 (32%) of the episodes presented in a coma.

**Table 1:** Baseline characteristics and outcome of episodes initially admitted to the ICU

Characteristic	ICU, *N* = 1369	Characteristic	ICU, *N* = 1369
Median age (IQR), years^a^	61 (49–69)	**Positive blood culture**	957/1197 (80%)
Female sex	632/1369 (46%)	Causative pathogen	
Symptoms < 24 h	637/1300 (49%)	*S. pneumoniae*	1,071/1369 (78%)
Recurrent meningitis	65/1367 (5%)	*N. meningitidis*	124/1369 (9%)
CSF leak	28/1364 (2%)	*L. monocytogenes*	36/1369 (3%)
Extrameningeal infection	656/1356 (48%)	*H. influenzae*	27/1,369 (2%)
Otitis or sinusitis	517/1317 (39%)	**Initial treatment**	
Endocarditis	26/1312 (2%)	Amoxicillin + third-generation cephalosporin	802/1367 (59%)
Pneumonia	141/1313 (11%)	Amoxicillin monotherapy	60/1367 (4%)
**Immunocompromised state**	458/1369 (33%)	Third-generation cephalosporin monotherapy	354/1,367 (26%)
Active cancer	65/1360 (5%)	Other antibiotic regimen	151/1367 (11%)
Diabetes	206/1363 (15%)	Adjunctive dexamethasone conform protocol	1134/1326 (86%)
HIV	11/1367 (1%)	**Radiological examination on admission**	
Asplenia	27/1,367 (2%)	Abnormal CT/MRI brain scan	602/1258 (48%)
Alcoholism	110/1,364 (8%)	Generalized oedema	134/1238 (11%)
Immunosuppressive treatment	120/1361 (9%)	Hydrocephalus	72/1241 (6%)
**Symptoms on admission**		Hypodensity (recent)	63/1234 (5%)
Headache	855/1,103 (78%)	Mastoid opacification	317/1200 (26%)
Nausea	572/1,050 (54%)	Sinus opacification	224/1189 (19%)
Neck stiffness	866/1216 (71%)	**Clinical course**	
Rash	123/1202 (10%)	Pneumonia	247/1268 (19%)
Median temperature (°C)^b^	38.9 (37.9–39.7)	Circulatory shock	220/1272 (17%)
Fever (> 38 °C)	979/1325 (74%)	Respiratory failure	470/1300 (36%)
Heart rate (beats/minute)^c^	100 (86–120)	Mechanical ventilation	715/1329 (54%)
Systolic blood pressure (mmHg)^d^	144 (125–164)	Seizures	230/1313 (18%)
Diastolic blood pressure (mmHg)^e^	80 (69–91)	Cerebral infarction	206/1258 (16%)
Glasgow Coma Scale Score^f^	10 (8–12)	Mono-, hemiparesis or aphasia	362/1,220 (30%)
Altered mental status (GCS < 14)	1,155/1360 (85%)	Sinus thrombosis	38/1,236 (3%)
Coma (GCS < 8)	431/1360 (32%)	**Outcome**	
Seizures	154/1,294 (12%)	Total duration of hospital stay (days)^p^	15 (12–24)
Babinski reflex	212/1109 (19%)	Days to death^q^	6 (2–14)
Cranial nerve palsy	97/1074 (9%)	Duration of ICU stay (days)^r^	3 (2–8)
Mono-, hemiparesis or aphasia	269/1163 (23%)	Glasgow Outcome Score	-
**Blood results**		1 (death)	304/1,369 (22%)
C-reactive protein (mg/L)^g^	204 (97–317)	2 (vegetative state)	4/1369 (0%)
Leukocytes (× 10^9/L)^h^	17 (12–23)	3 (severe disability)	77/1369 (6%)
Thrombocytes (× 10^9/L)^i^	190 (142–252)	4 (moderate disability)	258/1369 (19%)
Glucose (mmol/L)^j^	9.4 (7.7–11.9)	5 (mild or no disability)	726/1,369 (53%)
CSF chemistry		**Neurological sequelae**	
Opening pressure (cm H_2_O)^k^	48 (34–50)	Cognitive impairment	220/878 (25%)
Protein (g/L)^l^	4.4 (2.7–6.5)	Cranial nerve palsy	130/914 (14%)
Glucose (mmol/L)^m^	0.30 (0.10–2.20)	Mono-, hemiparesis or aphasia	74/934 (8%)
CSF:blood glucose ratio^n^	0.03 (0.01–0.23)	Hearing impairment	342/1028 (33%)
White cell count (per mm^3^)^o^	2850 (592–7595)	**Type of hospital**	
< 100 per mm^3^	150/1293 (12%)	Academic hospital	217/1369 (16%)
100–999 per mm3	263/1293 (20%)	Large non-academic hospital	628/1369 (46%)
> 999 per mm3	880/1293 (68%)	Regional hospital	524/1369 (38%)

Data presented as n/N (%) or median (IQR)

*CSF*   cerebrospinal fluid. *LP*   lumbar puncture. *ICU*  Intensive Care Unit. *Non-ICU ward*   Neurology ward, medium care unit, other

^a^Age was known in 1369 (100%) episodes. ^b^Temperature was known in 1323 (97%) episodes. ^c^Heart rate was known in 1310 (96%) episodes. ^d^Systolic blood pressure was known in 1314 (96%) episodes. ^e^Diastolic blood pressure was known in 1313 (96%) episodes. ^f^Glasgow Coma Scale score was known in 1360 (99%) episodes. ^g^C-reactive protein level was known in 1332 (97%) episodes. ^h^Blood leukocyte count was known in 1,353 (99%) episodes. ^i^Blood thrombocyte count was known in 1301 (95%) episodes. ^j^Blood glucose was known in 1,286 (94%) episodes. ^k^Opening pressure in CSF was known in 538 (39%) episodes. ^l^CSF protein level was known in 1270 (93%) episodes. ^m^CSF glucose level was known in 1286 (94%) episodes. ^n^CSF:blood glucose ratio was known in 1226 (90%) episodes. ^o^CSF white cell count was known in 1293 (94%) episodes. ^p^Duration of stay was known in 1027 (75%) episodes. Episodes that died were not included in this variable. ^q^Days to death was known in 297 (98%) episodes. ^r^Duration of ICU stay was known in 283 (21%) episodes

A lumbar puncture was performed in all episodes initially admitted to the ICU. The median CSF white cell count was 2850 per mm^3^ (IQR 592–7595). Cranial imaging was done on admission for 1258 of 1368 episodes (92%) and abnormalities were recorded in 602 of 1258 episodes (48%), most commonly sinus or mastoid opacification (514 of 1200 [45%]), generalized brain edema (134 of 1238 [11%]), and hydrocephalus (72 of 1241 episodes [6%]). Cranial imaging preceded lumbar puncture in 988 of 1106 episodes (89%) with neuroimaging data available. The most common causative pathogens were *S. pneumoniae* (1071 of 1369 [78%]) and *N. meningitidis* (124 of 1369 [9%]). Blood cultures were positive for 957 of 1197 episodes (80%) with cultures done. Initial antibiotic treatment included a combination of amoxicillin with a third-generation cephalosporin in 802 of 1367 episodes (59%), monotherapy third-generation cephalosporin in 354 of 1367 episodes (26%), and penicillin or amoxicillin monotherapy in 60 of 1367 episodes (4%). Other antibiotic regimens were used in 151 of 1357 episodes (11%). Adjunctive dexamethasone was administered in 1211 of 1326 episodes (91%), and according to protocol (defined as 4 times 10 mg a day, for 4 days) in 1134 of 1326 episodes (86%).

The clinical course of episodes initially admitted to the ICU was often complicated by respiratory failure (470 of 1300 [36%]), need for mechanical ventilation (715 of 1329 [54%]), seizures (230 of 1313 [18%]), and focal neurological deficits (362 of 1220 [30%]). An unfavourable outcome occurred in 643 of 1369 episodes (47%) and 304 (of 1369 [22%]) episodes died. The rate of unfavourable outcome was relatively stable over the years with two peaks between the periods 2009–2010 and 2020 (Additional file 1: Figure S1). In the year 2020, during the Covid-19 pandemic, the increase in unfavourable outcome co-existed with a decrease of the proportion of patients admitted to the ICU, suggesting that only the most severely ill patients were admitted to ICU. The overall case-fatality rate varied by the causative organism: 240 of 1071 episodes (23%) for pneumococcal meningitis, 6 of 124 episodes (5%) for meningococcal meningitis, 2 of 27 episodes (7%) for *Haemophilus influenzae* meningitis, 17 in 36 episodes (47%) for listeria meningitis and 10 of 17 (59%) for *Staphylococcus aureus* meningitis. Of the 304 non-survivors, the cause of death was known for 244 episodes (80%). Death was due to neurological complications in 148 of 244 episodes (61%) and to systemic complication in 96 of 244 episodes (39%). The most common causes of death were brain herniation (in 63 of 244 episodes [26%]), cerebrovascular complications (in 39 of 244 episodes [16%]), and withdrawal of care because of poor neurological prognosis (in 34 of 244 episodes [14%]).

A multivariable analysis assessing the independent predictors for initial ICU admission in the total cohort of bacterial meningitis episodes (*N* = 2709) showed that younger age (< 70 years), male sex, immunocompromised state, presence of rash, low Glasgow Coma Scale score, seizures on admission, heart rate above 100 beats per minute, high temperature, low thrombocyte count in blood, high protein in CSF and pneumococcal meningitis were predictive for ICU admission (Additional file 2: Table S2).

A multivariable analysis including only the episodes initially admitted to the ICU (*N* = 1369) revealed several characteristics independently associated with unfavourable outcome (Table 2): admittance to an academic hospital, advanced age (> 70 years), alcoholism, cranial nerve palsies or seizures on admission, low leukocyte count in blood, high C-reactive protein in blood, low CSF:blood glucose ratio, listerial meningitis, need for mechanical ventilation, circulatory shock and persistent fever.

**Table 2:** Factors associated with an unfavourable outcome in the episodes initially admitted to the intensive care unit (*N* = 1369)

	Favourable outcome (*N* = 726)	Unfavourable outcome (*N* = 643)	Univariable OR unfav outcome (95%CI)	Multivariable OR unfav. outcome (95%CI)	*p*-value multivariable analysis
Type of hospital					
Academic hospital	88/726 (12%)	129/643 (20%)	1.6 (1.2–2.2)*	1.59 (1.04–2.43)	0.03
Large non-academic hospital	327/726 (45%)	301/643 (47%)	*Reference*	–	–
Regional hospital	311/726 (43%)	213/643 (33%)	0.7 (0.6–0.9)*	0.79 (0.58–1.08)	0.145
Age (years)^a^	59 (45–66)	63 (54–72)	–	–	–
16 – 39	140/718 (19%)	49/637 (8%)	0.4 (0.3–0.6)*	0.59 (0.37–0.96)	0.03
40–70	483/718 (67%)	394/637 (62%)	*Reference*	–	–
> 70	95/718 (13%)	194/637 (30%)	2.5 (1.9–3.3)*	2.86 (1.98–4.14)	< 0.001
Female sex	337/726 (46%)	295/643 (46%)	1.0 (0.8–1.2)	1.01 (0.76–1.36)	0.925
Otitis and/or sinusitis on admission	316/709 (45%)	201/608 (33%)	0.6 (0.5–0.8)*	0.75 (0.54–1.04)	0.08
Immunocompromised state	207/726 (29%)	251/643 (39%)	1.6 (1.3–2.0)*	–	–
Active cancer	27/723 (4%)	38/637 (6%)	1.7 (1.0–2.7)	0.67 (0.32–1.40)	0.288
Diabetes mellitus	97/725 (13%)	109/638 (17%)	1.3 (1.0–1.8)	1.13 (0.74–1.73)	0.568
HIV	6/726 (1%)	5/641 (1%)	1.2 (0.4–3.6)	0.75 (0.16–3.44)	0.707
Asplenia	12/726 (2%)	15/641 (2%)	1.5 (0.7–3.1)	1.31 (0.49–3.48)	0.594
Alcoholism	34/725 (5%)	76/639 (12%)	2.7 (1.8–4.2)*	1.71 (1–2.95)	0.05
Immunosuppressive drugs	58/725 (8%)	62/636 (10%)	1.2 (0.9–1.8)	0.87 (0.50–1.52)	0.627
Clinical signs and symptoms					
Triad**	342/673 (51%)	243/581 (42%)	0.7 (0.6–0.9)*	1.02 (0.74–1.40)	0.900
Rash	80/651 (12%)	43/551 (8%)	0.6 (0.4–0.9)*	0.92 (0.51–1.68)	0.789
Seizures	60/703 (9%)	94/591 (16%)	2.1 (1.5–2.9)*	1.57 (1–2.52)	0.05
Cranial nerve palsy	30/603 (5%)	67/471 (14%)	3.6 (2.4–5.4)*	2.76 (1.61–4.73)	< 0.001
Mono-, hemiparesis or aphasia	131/657 (20%)	138/506 (27%)	2.2 (1.6–3.0)*	1.28 (0.82–1.99)	0.280
Glasgow Coma Scale Score^b^	10 (9–13)	9 (7–11)	0.9 (0.8–0.9)*	0.96 (0.90–1.02)	0.136
Systolic blood pressure(mmHg)^c^	142 (124–162)	145 (125–165)	1.00 (1.00–1.01)	–	–
Heart rate (bpm)^d^	99 (84–110)	106 (90–125)	–	–	–
< 60	20/702 (3%)	9/608 (1%)	0.7 (0.3–1.5)	0.57 (0.20–1.66)	0.304
60–100	378/702 (54%)	259/608 (43%)	*Reference*	–	–
> 100	304/702 (43%)	340/608 (56%)	1.6 (1.3–2.1)*	1.21 (0.89–1.64)	0.217
Temperature (°C)^e^	39.0 (38.1–39.7)	38.8 (37.6–39.6)	0.8 (0.8–0.9)*	0.90 (0.80–1.02)	0.097
Blood results					
Leukocytes (× 10^9^ cells/l)^f^	18 (13–23)	16 (10–22)	–	–	–
< 4.00	6/719 (1%)	35/634 (6%)	4.4 (1.8–10.8)*	4.16 (1.34–12.93)	0.01
4.00–10.00	87/719 (12%)	116/634 (18%)	*Reference*	–	–
> 10.00	626/719 (87%)	483/634 (76%)	0.6 (0.4–0.8)*	0.93 (0.61–1.42)	0.744
C-reactive protein (per 10 mg/L)^g^	16 (8–27)	25 (14–36)	1.04 (1.03–1.05)*	1.03 (1.02–1.04)	< 0.001
CSF results					
Leukocytes in CSF (per µL)^h^	4,219 (1190–9302)	1,567 (252–5,920)	–	–	–
< 100	44/690 (6%)	104/603 (17%)	1.8 (1.2–2.8)*	1.18 (0.65–2.15)	0.584
100–999	111/690 (16%)	154/603 (26%)	*Reference*	–	–
> 999	535/690 (78%)	345/603 (57%)	0.5 (0.4–0.6)*	0.74 (0.49–1.11)	0.140
CSF:blood glucose ratio^i^	0.07 (0.01–0.29)	0.02 (0.01–0.12)	–	–	–
< 0.25	467/657 (71%)	472/555 (85%)	1.5 (0.9–2.6)	2.37 (1.01–5.53)	0.05
0.26–0.5	159/657 (24%)	63/555 (11%)	0.6 (0.3–1.2)	1.14 (0.47–2.74)	0.775
> 0.5	31/657 (5%)	20/555 (4%)	*Reference*	–	–
Protein (per 10 g/L)^j^	0.39 (0.25–0.60)	0.49 (0.30–0.69)	1.00 (0.98–1.01)	–	–
Causative pathogen					
* S. pneumoniae*	554/726 (76%)	517/643 (80%)	*Reference*	–	–
* N. meningitidis*	97/726 (13%)	27/643 (4%)	0.3 (0.2–0.5)*	0.58 (0.30–1.12)	0.103
* H. influenzae*	19/726 (3%)	8/643 (1%)	0.5 (0.2–1.0)	0.41 (0.12–1.35)	0.142
* L. monocytogenes*	10/726 (1%)	26/643 (4%)	2.8 (1.3–5.8)*	4.51 (1.67–12.17)	0.003
Other	46/726 (6%)	65/643 (10%)	1.5 (1.0–2.3)*	1.85 (1.04–3.28)	0.04
Treatment and complications					
Adjunctive dexamethasone	639/711 (90%)	507/614 (83%)	0.5 (0.4–0.7)*	0.77 (0.48–1.23)	0.271
Mechanical ventilation	255/707 (36%)	460/622 (74%)	4.9 (3.9–6.1)*	2.83 (2.04–3.94)	< 0.001
Persistent fever^k^	36/690 (5%)	109/571 (19%)	4.3 (2.9–6.5)*	2.13 (1.31–3.47)	0.002
Circulatory shock	40/688 (6%)	180/584 (31%)	7.3 (5.1–10.5)*	4.70 (2.90–7.63)	< 0.001

Data presented as n/N (%) or median (IQR)

*CSF*  cerebrospinal fluid, *ICU*  Intensive Care Unit

*Factor is significant in univariate analysis. **Triad defined as neck stiffness, fever and altered mental status on admission

^a^Age was known in 726 (100%) episodes with a favourable and 643 (100%) with an unfavourable outcome. ^b^Glasgow Coma Scale Score was known in 722 (99%) episodes with a favourable and 638 (99%) with an unfavourable outcome. ^c^Systolic blood pressure was known in 703 (97%) episodes with a favourable and 611 (95%) with an unfavourable outcome. ^d^Heart rate was known in 702 (97%) episodes with a favourable and 608 (95%) with an unfavourable outcome. ^e^Temperature was known in 709 (98%) episodes with a favourable and 614 (95%) with an unfavourable outcome. ^f^Blood leukocyte count was known in 719 (99%) episodes with a favourable and 634 (99%) with an unfavourable outcome. ^g^C-reactive protein level was known in 710 (98%) episodes with a favourable and 622 (97%) with an unfavourable outcome. ^h^CSF leukocyte count was known in 690 (95%) episodes with a favourable and 603 (94%) with an unfavourable outcome. ^i^ CSF:blood glucose ratio was known in 661 (91%) episodes with a favourable and 565 (88%) with an unfavourable outcome. ^j^CSF protein level was known in 674 (93%) episodes with a favourable and 596 (93%) with an unfavourable outcome. ^k^Persistent fever was defined as fever lasting for longer than 24 h after administration of antibiotics

Of the 1340 episodes initially not admitted to the ICU, 204 (15%) were transferred secondarily to the ICU (Fig. 1) after a median of 1 day (IQR 0–3 days); 74 out of 190 (39%) episodes were transferred on the same day and 141 of 190 (74%) within 3 days (Table 3). Reasons indicated by physicians for ICU transfer were neurological complications in 80 of 189 episodes (42%), mostly seizures (22 of 189 [12%]), respiratory failure in 63 of 189 episodes (33%) and haemodynamic instability in 25 of 189 episodes (1%). The most common causative pathogens were *S. pneumoniae* (135 of 204 [66%]) and *L. monocytogenes* (31 of 204 [15%]; Additional file 2: Table S3). The rate of unfavourable outcome (135 of 204 [66%]) as well as mortality (30 of 204 [30%]) were high in the transfer cohort. The most common cause of death in episodes secondarily transferred to the ICU was haemodynamic instability or respiratory failure (10 of 50 [20%]). Interestingly, 129 of 1331 episodes not admitted to the ICU initially presented with a Glasgow Coma Scale score less than 8. Of these 129 patients, 5 patients (3%) died on the emergency room before they even could be admitted to the ICU, 16 patients (12%) were not transferred to the ICU because of treatment limitations based on advanced age or pre-existing medical conditions, and 33 episodes (31%) quickly improved in the emergency room (for example because of epileptic seizure). For 75 patients the reason for admission to a non-ICU ward despite their low Glasgow Coma Scale score was unclear.

**Table 3:** Duration of stay on non-ICU ward until transfer to ICU, and primary reason for ICU transfer

Time until ICU transfer	
Days until transfer to ICU^a^	1 (0–3)
Transfer same day	74/190 (39%)
Transfer < 3 days	141/190 (74%)
Primary reason for ICU transfer	
Observation	4/189 (2%)
Neurological complications	80/189 (42%)
Sedation due to motor agitation	10/189 (5%)
Seizures	22/189 (12%)
Cerebrovascular complications	8/189 (4%)
Ischaemic stroke	2/8 (30%)
Intracranial haemorrhage	3/8 (38%)
Cerebral vasculitis or delayed thrombosis	3/8 (38%)
Hydrocephalus	6/189 (3%)
Generalized cerebral edema	3/189 (2%)
Deterioration of consciousness, not further specified	28/189 (15%)
Otherwise neurological	3/189 (2%)
Haemodynamic complications	35/189 (19%)
Haemodynamic instability	25/189 (13%)
Otherwise haemodynamic	10/189 (5%)
Respiratory failure	63/189 (33%)
Post-operative	7/189 (4%)
Neurosurgical	3/7 (43%)
Non-neurosurgical	4/7 (57%)
Unknown	15/204 (7%)

Data presented as n/N (%) or median (IQR)

^a^Days until transfer to ICU is known for 190 of 204 episodes. Reason of transfer to ICU is known for 189 of 204 episodes

## Discussion

Our study shows that a substantial proportion of bacterial meningitis cases (51%) are initially admitted to the ICU. A similar proportion of bacterial meningitis patients who required ICU admission was found in a recent UK study reporting (53%) but is lower than that reported in French cohort study (79%) [25, 26]. Furthermore, we found that the Covid-19 pandemic led to a decrease in the rate of ICU admission which was associated with a higher likelihood of unfavourable outcome. Indeed, an observational cohort of 130,698 patients in the UK showed that for patients admitted to ICU during the pandemic, levels of ICU capacity strain were associated with higher mortality even after accounting for differences in baseline characteristics [27].

The rate of unfavourable outcome (47%) and mortality (22%) was high amongst bacterial meningitis patients admitted to the ICU. We found several characteristics predictive of unfavourable outcome. First, admission to a tertiary care facility (academic hospital) was associated with unfavourable outcome, consistent with previous research that suggests that patients admitted in ICU tertiary care facilities have a poorer outcome compared to those treated in lower-level healthcare settings [28]. Second, patient factors such as advanced age and alcoholism were associated with unfavourable outcomes, which is consistent with prior findings [1, 2, 29, 30]. Third, established factors predictive of severe illness such as seizures, cranial nerve palsy, low leukocyte count in blood, high C-reactive protein in blood, low CSF glucose concentration, need for mechanical ventilation, and presence of circulatory shock were also found to be predictors of unfavourable outcome [2, 29, 31, 32]. Finally, we also identified persistent fever and infection with *L. monocytogenes* as predictors of unfavourable outcome. Persistent fever has been previously reported as a predictive factor for unfavourable outcome in a meta-analysis of data from children bacterial meningitis [33]. *L. monocytogenes* can cause bacterial meningitis in immunocompromised individuals and the elderly [34, 35], and has been linked to high rates of unfavourable outcome previously [36, 37]. We now show that on the ICU, listerial meningitis remains an independent predictor of unfavourable outcome in a model corrected for baseline characteristics, factors predictive for severe illness and persistent fever. Previous studies showed that delayed initiation of antibiotic treatment is also associated with increased in-hospital mortality and unfavourable outcome, [10] but unfortunately, as the exact timing of antibiotic treatment (in hours) is not known in our study, we could not correct for this in our multivariable analysis.

We also identified characteristics predictive of initial ICU admission, both patient factors (such as age younger than 70 years and immunocompromised state), and factors indicative for severe (pneumococcal) disease and systemic compromise (such as presence of rash, seizures on admission, impaired consciousness, high fever, thrombocytopenia and high protein levels in CSF). Interestingly, female patients had a lower likelihood of being admitted to the ICU. In our study, this effect remained robust in a multivariable analysis that corrected for severity. The decision to admit a meningitis patient to the ICU is typically based on the severity of their medical condition and the need for close monitoring and specialized care [16], rather than sex. However, hospital-population based studies have shown that men are generally more frequently admitted to ICUs, utilize more ICU resources, and are more likely to receive advanced life-supporting measures compared to women [38, 39]. In 2021, a retrospective analysis was conducted on 450,948 adult patients with neuro- and cardiovascular diseases who were admitted to various hospitals in Switzerland [40]. The findings of this analysis suggested that women had a reduced probability of receiving ICU treatment, regardless of the severity of their illness. In bacterial meningitis male sex has been identified as a predictive factor for poor outcomes both in children and adults [26, 39, 41]. Outcome in women tends to be better than for men, even though females tend to exhibit higher disease severity and elevated inflammation markers upon admission [41]. This observation might be partially attributed to the more favourable response of females to anti-inflammatory treatment involving corticosteroids [42]. Additionally, sex steroid hormones could potentially play a role in these disparities [43].

Of the 1340 episodes (49%) that were initially not admitted to the ICU, 204 episodes (15%) were still transferred to the ICU later on during admission. Interestingly the median duration until ICU transfer was only 1 day, showing that a substantial proportion of bacterial meningitis episodes that were initially not admitted to the ICU clinically deteriorated within only a few hours after admission to a non-ICU ward. Systemic complications in general, and specifically cardiorespiratory failure, were the main listed reasons for transfer to the ICU. Furthermore, both unfavourable outcome and mortality were extremely high in the transfer cohort (respectively, 66% and 30%), even compared to the cohort of patients initially admitted to the ICU. These findings raise the question to whether a more liberate ICU admission policy on initial presentation could have improved outcome, as early recognition of circulatory shock and respiratory failure is crucial for effective treatment of these complications. Even though it remains difficult to specify on an individual level whether the severity of the patients included in the transfer cohort was underestimated in the Emergency Department, the results of this study further substantiate the recommendation towards a more liberate ICU approach including respiratory and/or circulatory support for bacterial meningitis patients [17].

Our study has important limitations. First, only patients who underwent lumbar puncture were included in the study. Therefore, patients with absolute contraindications for a lumbar puncture, such as severe thrombocytopenia in sepsis or intracranial complications with risk of brain herniation, are not included. Second, our study in an observational study. Associations between the Covid-19 pandemic, ICU admission, and outcome have been observed and make sense, but the study design precludes conclusions about causality. Furthermore, our database lacks extensive data on ICU parameters such as respiratory rate, lactate levels and vasopressor need, precluding us from an objective assessment of septic shock or ICU admission criteria. Nevertheless, our prospective nationwide cohort study including more than 2,700 patients is the most comprehensive cohort study on bacterial meningitis to date. Third, our data only include outcome data scored at hospital discharge, which is relatively early for neurological assessment of disability. Fourth, although we prospectively collected data on complications and ICU admission, the rationale of physicians to admit to the ward, a specialized care unit, or ICU remain unclear to some extent. Furthermore, due to the observational nature of our cohort, our database lacks clear criteria and comprehensive details regarding the management of patients during their ICU stay.

In conclusion, monitoring and/or treatment in an ICU is performed in the majority of adults with community-acquired bacterial meningitis. We observed that the likelihood of ICU admission decreased during the start of the Covid-19 pandemic, which was associated with an increased rate of unfavourable outcome. The majority of meningitis patients admitted on the ICU will need mechanical ventilation, but ICU monitoring is also important to recognize changes in the patient’s consciousness and the development of new neurologic signs, monitor for subtle seizures, and treat severe agitation effectively [16]. Bacterial meningitis is often associated with circulatory shock, which is an important predictor of outcome [1, 2], requiring aggressive supportive treatment [3, 44].

### Supplementary Information

**Additional file 1: Figure S1.** Proportion of bacterial meningitis episodes initially admitted to the intensive care unit (ICU; blue line), and the proportion of unfavourable outcome (red line) per year (+ 95% confidence interval). Year is calculated as epidemiological year (from June to July). Unfavourable outcome is defined as Glasgow Outcome Scale score between 1 and 4. Significant decrease in ICU proportion in 2020 (*p* = 0.004), trend towards an increased proportion of unfavourable outcome (*p* = 0.17).

**Additional file 2: Table S1.** Comparison of baseline and outcome characteristics of the initial ICU cohorts of 2020 versus 2015-2019. **Table S2**. Prognostic factors for initial intensive care unit (ICU) admission in all bacterial meningitis episodes (*N* = 2709). **Table S3**. Baseline and clinical characteristics of episodes transferred to ICU later during admission (*N* = 204).

## Data Availability

The data that support the findings of this study are available upon request from any qualified investigator from the corresponding author [DvdB].

## Abbreviations

ICU: Intensive care unit
NRLBM: Netherlands reference laboratory for bacterial meningitis
GOS: Glasgow Outcome Scale
GCS: Glasgow Coma Scale
HABM: Hospital-acquired bacterial meningitis
CSF: Cerebrospinal fluid
PCR: Polymerase chain reaction
