# Supplementary figures and images for: Characteristics and prognostic factors of bacterial meningitis in the intensive care unit: a prospective nationwide cohort study
Source: Ann Intensive Care. 2023 Dec 6;13:124. doi: 10.1186/s13613-023-01218-6 (PMC10700277; doi:10.1186/s13613-023-01218-6)

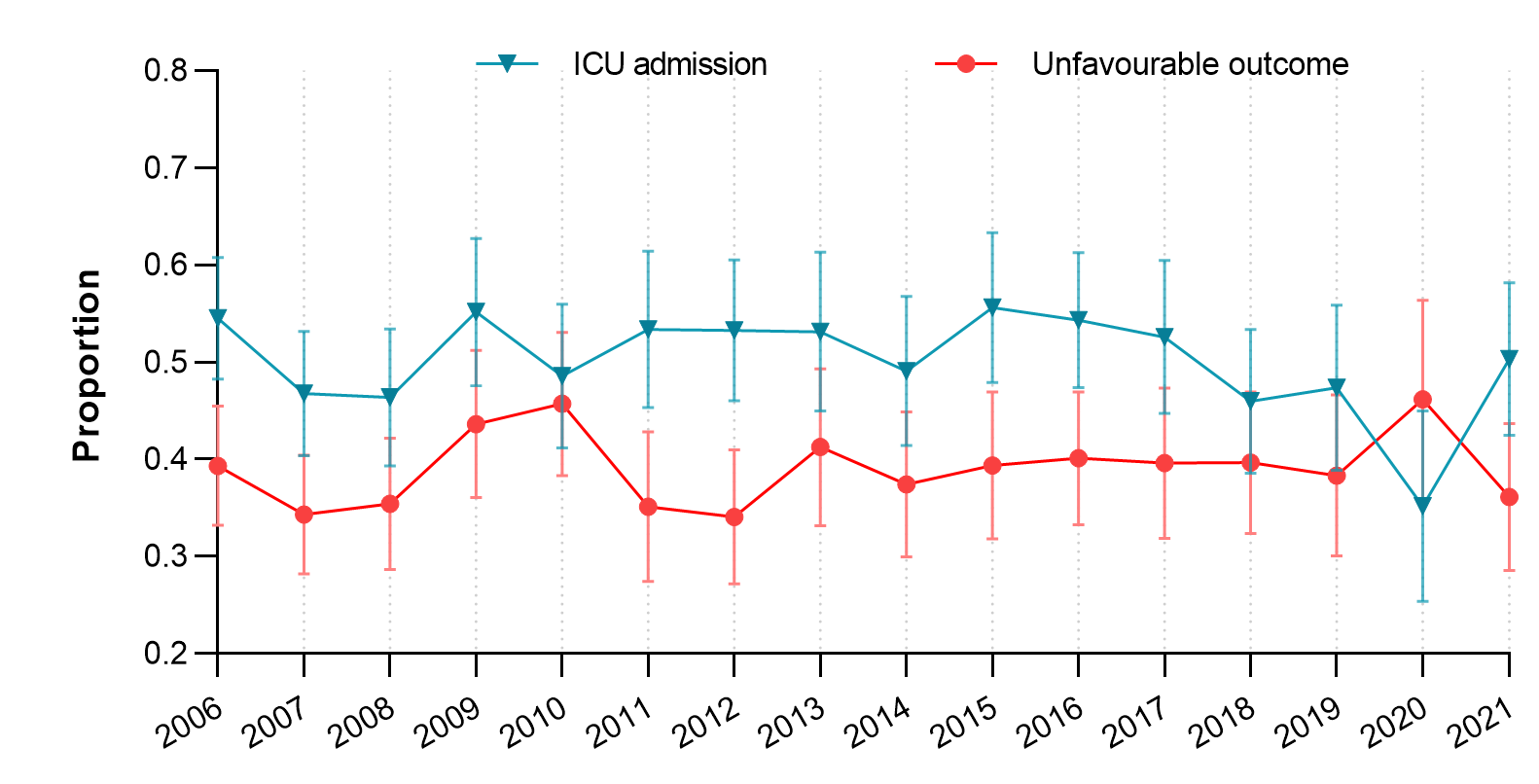

Supplement: Supplementary file 1 — Additional file 1: Figure S1. Proportion of bacterial meningitis episodes initially admitted to the intensive care unit (ICU; blue line), and the proportion of unfavourable outcome (red line) per year (+ 95% confidence interval). Year is calculated as epidemiological year (from June to July). Unfavourable outcome is defined as Glasgow Outcome Scale score between 1 and 4. Significant decrease in ICU proportion in 2020 (p = 0.004), trend towards an increased proportion of unfavourable outcome (p = 0.17). [file 13613_2023_1218_MOESM1_ESM.tif]
