# Supplementary material for: Characteristics and prognostic factors of bacterial meningitis in the intensive care unit: a prospective nationwide cohort study
Source: Ann Intensive Care. 2023 Dec 6;13:124. doi: 10.1186/s13613-023-01218-6 (PMC10700277; doi:10.1186/s13613-023-01218-6)
Supplement: Supplementary file 2 — Additional file 2: Table S1. Comparison of baseline and outcome characteristics of the initial ICU cohorts of 2020 versus 2015-2019. Table S2. Prognostic factors for initial intensive care unit (ICU) admission in all bacterial meningitis episodes (N = 2709). Table S3. Baseline and clinical characteristics of episodes transferred to ICU later during admission (N = 204). [file 13613_2023_1218_MOESM2_ESM.docx]

**Additional file 2: Tables**

**Additional file 2: Table S1** Comparison of baseline and outcome characteristics of the initial ICU cohorts of 2020 versus 2015-2019

| **Characteristic** | **2020** (N = 91) | **2015 - 2019** (N = 818) | **P-value** |
| --- | --- | --- | --- |
| Median age (IQR), years | 62 (48-70)^a^ | 62 (50-71)^b^ | 0.99 |
| Female sex | 35/91 (38%) | 402/818 (49%) | 0.06 |
| Immunocompromised state | 36/91 (40%) | 254/817 (31%) | 0.12 |
| Heart rate (beats per minute) | 102 (85-120)^c^ | 96 (81-111)^d^ | 0.05 |
| Temperature (°C) | 38.7 (37.6-39.6)^e^ | 38.8 (37.8-39.5)^f^ | 0.53 |
| Glasgow Coma Scale Score | 12 (9-14)^g^ | 11 (9-14)^h^ | 0.40 |
| Altered mental status | 59/90 (66%) | 548/810 (68%) | 0.72 |
| Coma | 17/90 (19%) | 158/810 (20%) | 1.00 |
| Cranial nerve palsy | 7/77 (9%) | 40/631 (6%) | 0.34 |
| CSF white cell count per mm^3^ | 2779 (898-6478)^i^ | 2181 (592-6132)^j^ | 0.22 |
| <100 per mm^3^ | 5/91 (5%) | 68/762 (9%) | - |
| 100-999 per mm3 | 19/91 (21%) | 168/762 (22%) | - |
| >999 per mm3 | 67/91 (74%) | 526/762 (69%) | - |
| **Causative pathogen** | - | - | 0.085 |
| *Streptococcus pneumoniae* | 58/91 (64%) | 539/818 (66%) | - |
| *Neisseria meningitidis* | 6/91 (7%) | 95/818 (12%) | - |
| *Listeria monocytogenes* | 13/91 (14%) | 61/818 (7%) | - |
| Other | 14/91 (15%) | 123/818 (15%) | - |
| Initial admission on ICU | 32/91 (35%) | 420/818 (51%) | 0.004 |
| Unfavourable outcome^g^ | 42/91 (46%) | 313/818 (38%) | 0.17 |

Data presented as n/N (%) or median (IQR). CSF = cerebrospinal fluid. ICU = intensive care unit. Unfavourable outcome is defined as Glasgow Outcome Scale score of 1-4. Altered menatal status is defined as a Glasgow Coma Scale Score of below 14. Coma is defined as a Glasgow Coma Scale Score of 8 or lower. Statistical differences were assessed using the Fisher’s exact test (2x2), the Chi-square test (more than 2x2) or the Mann-Whitney U test.

^a^Age was known for all episodes. ^b^Age was known for all episodes. ^c^Heart rate was known for 89 episodes. ^d^Heart rate was known for 757 episodes. ^e^Temperature was known for 87 episodes. ^f^Temperature was known for 774 episodes. ^g^Glasgow Outcome Scale score was known for 90 episodes. ^h^Glasgow Outcome Scale score was known for 810 episodes. ^i^CSF white cell count was known for 91 episodes. ^j^CSF white cell count was known for 763 episodes.

**Additional file S2: Table 2** Prognostic factors for initial intensive care unit (ICU) admission in all bacterial meningitis episodes (N = 2,709)

|  | **Initial ICU admission**  (N = 1,369) | **Initial admission non-ICU ward**  (N = 1,340) | **Univariable OR**  **(95%CI)** | **Multivariable OR (95%CI)** | **p-value**  **multi-variable**  **analysis** |
| --- | --- | --- | --- | --- | --- |
| Age (years)^a^ | 61 (49-69) | 61 (47-71) | … | … | … |
| 16 – 39 | 189/1,355 (14%) | 225/1,329 (17%) | 0.7 (0.6-0.9)* | 1.18 (0.90-1.56) | 0.238 |
| 40-70 | 877/1,355 (65%) | 758/1,329 (57%) | *Reference* | … | … |
| >70 | 289/1,355 (21%) | 346/1,329 (26%) | 0.7 (0.6-0.9)* | 0.67 (0.54-0.83) | <0.001 |
| Female sex | 632/1,369 (46%) | 704/1,340 (53%) | 0.8 (0.7-0.9)* | 0.74 (0.62-0.88) | <0.001 |
| Recurrent CABM | 65/1,367 (5%) | 106/1,339 (8%) | 0.6 (0.4-0.8)* | 1.03 (0.69-1.55) | 0.871 |
| CSF leak | 28/1,364 (2%) | 64/1,313 (5%) | 0.4 (0.3-0.6)* | 0.61 (0.5-1.08) | 0.09 |
| Symptoms <24 hours | 637/1,300 (49%) | 588/1,296 (45%) | 1.1 (1.0-1.3) | … | … |
| Predisposing otitis and/or sinusitis | 517/1,317 (39%) | 441/1,295 (34%) | 1.2 (1.1-1.5)* | 1.08 (0.89-1.32) | 0.435 |
| Predisposing pneumonia | 141/1,313 (11%) | 114/1,290 (9%) | 1.2 (1.0-1.6) | … | … |
| **Immunocompromised state** | 458/1,369 (33%) | 364/1,339 (27%) | 1.3 (1.1-1.6)* | 1.30 (1.06-1.59) | 0.01 |
| Active cancer | 65/1,360 (5%) | 83/1,332 (6%) | 0.8 (0.5-1.0) | … | … |
| Diabetes mellitus | 206/1,363 (15%) | 159/1,327 (12%) | 1.3 (1.1-1.7)* | … | … |
| HIV | 11/1,367 (1%) | 7/1,332 (1%) | 1.3 (0.5-3.4) | … | … |
| Asplenia | 27/1,367 (2%) | 23/1,336 (2%) | 1.1 (0.6-2.0) | … | … |
| Alcoholism | 110/1,364 (8%) | 57/1,335 (4%) | 2.0 (1.4-2.7)* | … | … |
| Immunosuppressive drugs | 120/1,361 (9%) | 133/1,329 (10%) | 0.9 (0.7-1.1) | … | … |
| **Clinical signs & symptoms** |  |  |  |  |  |
| Headache | 855/1,103 (78%) | 981/1,198 (82%) | 0.8 (0.6-1.0)* | … | … |
| Nausea | 572/1,050 (54%) | 694/1,151 (60%) | 0.8 (0.7-0.9)* | … | … |
| Neck stiffness | 866/1,216 (71%) | 906/1,244 (73%) | 0.9 (0.8-1.1) | … | … |
| Triad** | 585/1,254 (47%) | 394/1,286 (31%) | 2.0 (1.7-2.3)* | … | … |
| Rash | 123/1,202 (10%) | 92/1,193 (8%) | 1.3 (1.0-1.7) | 2.02 (1.42-2.88) | <0.001 |
| Seizures | 154/1,294 (12%) | 63/1,301 (5%) | 2.6 (1.9-3.6)* | 1.50 (1.07-2.12) | 0.02 |
| Cranial nerve palsy | 97/1,074 (9%) | 86/1,205 (7%) | 1.4 (1.0-1.9)* | 1.25 (0.87-1.79) | 0.235 |
| Mono-, hemiparesis or aphasia | 269/1,163 (23%) | 271/1,256 (22%) | 1.9 (1.5-2.3)* | 0.95 (0.73-1.23) | 0.674 |
| Babinski reflex | 212/1,109 (19%) | 142/1,170 (12%) | 1.7 (1.3-2.1)* | 1.09 (0.81-1.46) | 0.581 |
| Glasgow Coma Scale Score^b^ | 10 (8-12) | 13 (10-15) | 0.8 (0.7-0.8)* | … | … |
| GCS 14-15 | 205/1,360 (15%) | 604/1,331 (45%) | *Reference* | … | … |
| GCS <14 | 724/1,360 (53%) | 598/1,331 (45%) | 3.6 (2.9-4.3)* | 2.71 (2.11-3.48) | <0.001 |
| GCS <8 | 431/1,360 (32%) | 129/1,331 (10%) | 9.8 (7.6-12.6)* | 5.96 (4.27-8.31) | <0.001 |
| Systolic blood pressure(mmHg)^c^ | 144 (125-164) | 142 (125-160) | 1.001 (0.998-1.004) | … | … |
| Heart rate (bpm)^d^ | 100 (86-120) | 96 (82-109) | … | … | … |
| <60 | 29/1,310 (2%) | 27/1,269 (2%) | 1.2 (0.7-2.1) | 1.23 (0.56-2.33) | 0.519 |
| 60-100 | 637/1,310 (49%) | 759/1,269 (60%) | *Reference* | … | … |
| >100 | 644/1,310 (49%) | 483/1,269 (38%) | 1.6 (1.4-1.9)* | 1.27 (1.05-1.53) | 0.01 |
| Temperature (°C) ^e^ | 38.9 (37.9-39.7) | 38.8 (37.7-39.5) | 1.1 (1.0-1.1) | 1.08 (1.00-1.16) | 0.05 |
| Fever (>38°C) | 979/1,325 (74%) | 926/1,299 (71%) | 1.1 (1.0-1.4) | … | … |
| **Blood results** |  |  |  |  |  |
| Leukocytes (x10^9^ cells/liter)^f^ | 17 (12-23) | 16 (12-22) | … | … | … |
| <4.00 | 41/1,353 (3%) | 27/1,313 (2%) | 1.4 (0.8-2.3) | 1.25 (0.69-2.29) | 0.464 |
| 4.00-10.00 | 203/1,353 (15%) | 192/1,313 (15%) | *Reference* | … | … |
| >10.00 | 1,109/1,353 (82%) | 1,094/1,313 (83%) | 1.0 (0.8-1.2) | 1.08 (0.83-1.40) | 0.581 |
| Thrombocytes (x10^9^ cells/liter)^g^ | 190 (142-252) | 207 (159-260) | … | … | … |
| <150 | 392/1,301 (30%) | 270/1,248 (22%) | 1.5 (1.3-1.8)* | 1.47 (1.19-1.82) | <0.001 |
| 151-450 | 879/1,301 (68%) | 953/1,248 (76%) | *Reference* | … | … |
| >450 | 30/1,301 (2%) | 25/1,248 (2%) | 1.3 (0.8-2.2) | 1.32 (0.72-2.42) | 0.367 |
| C-reactive protein (per 10 mg/L)^h^ | 20 (10-32) | 16 (7-28) | 1.02 (1.01-1.02)* | 1.01 (1.00-1.01) | 0.07 |
| Glucose (mmol/L)^i^ | 9.4 (7.7-11.9) | 8.7 (7.3-10.6) | 1.09 (1.06-1.12)* | … | … |
| **CSF results** |  |  |  |  |  |
| Leukocytes in CSF (per µL)^j^ | 2,850 (592-7,595) | 2,194 (587-5,646) | … | … | … |
| <100 | 148/1,293 (11%) | 114/1,294 (9%) | 1.5 (1.1-2.0)* | 1.14 (0.81-1.60) | 0.467 |
| 100-999 | 265/1,293 (20%) | 307/1,294 (24%) | *Reference* | … | … |
| >999 | 880/1,293 (68%) | 873/1,294 (67%) | 1.2 (1.0-1.4) | 0.95 (0.75-1.20) | 0.676 |
| CSF:blood glucose ratio^k^ | 0.03 (0.01-0.23) | 0.10 (0.01-0.31) | … | … | … |
| <0.25 | 939/1,212 (77%) | 810/1,185 (68%) | 1.8 (1.2-2.5)* | 0.99 (0.64-1.55) | 0.975 |
| 0.26-0.5 | 222/1,212 (18%) | 304/1,185 (26%) | 1.1 (0.8-1.7) | 1.05 (0.65-1.70) | 0.843 |
| >0.5 | 51/1,212 (4%) | 71/1,185 (6%) | *Reference* | … | … |
| Protein (per 10 g/L)^l^ | 0.44 (0.27-0.65) | 0.35 (0.19-0.57) | 2.04 (1.63-2.56)* | 1.54 (1.22-1.94) | <0.001 |
| **Causative pathogen** |  |  |  |  |  |
| *S. pneumoniae* | 1,071/1,369 (78%) | 812/1,340 (61%) | *Reference* | … | … |
| *N. meningitidis* | 124/1,369 (9%) | 156/1,340 (12%) | 0.6 (0.5-0.8)* | 0.76 (0.53-1.08) | 0.127 |
| *H. influenzae* | 27/1,369 (2%) | 82/1,340 (6%) | 0.3 (0.2-0.4)* | 0.44 (0.26-0.75) | 0.002 |
| *L. monocytogenes* | 36/1,369 (3%) | 133/1,340 (10%) | 0.2 (0.1-0.3)* | 0.23 (0.15-0.37) | <0.001 |
| Other | 111/1,369 (8%) | 157/1,340 (12%) | 0.5 (0.4-0.7)* | 0.73 (0.54-0.99) | 0.04 |
| Positive blood culture | 957/1,197 (80%) | 832/1,122 (74%) | 1.4 (1.2-1.7)* | 1.01 (0.80-1.26) | 0.964 |

Data presented as n/N (%) or median (IQR). CSF = cerebrospinal fluid. LP = lumbar puncture. ICU = Intensive Care Unit. *Factor is significant in univariate analysis.

**Triad defined as neck stiffness, fever and altered mental status on admission.

^a^Age was known in 1,369 (100%) episodes admitted to ICU and 1,340 (100%) admitted to other wards. ^b^Glasgow Coma Scale Score was known in 1,360 (99%) episodes admitted to ICU and 1,331 (99%) admitted to other wards. ^c^Systolic blood pressure was known in 1,314 (96%) episodes admitted to ICU and 1,291 (96%) admitted to other wards. ^d^Heart rate was known in 1,310 (96%) episodes admitted to ICU and 1,269 (95%) admitted to other wards. ^e^Temperature was known in 1,323 (97%) episodes admitted to ICU and 1,299 (97%) admitted to other wards. ^f^ Blood leukocyte count was known in 1,353 (99%) episodes admitted to ICU and 1,313 (98%) admitted to other wards. ^g^Blood thrombocyte count was known in 1,301 (95%) episodes admitted to ICU and 1,248 (91%) admitted to other wards. ^h^C-reactive protein level was known in 1,332 (97%) episodes admitted to ICU and 1,293 (94%) admitted to other wards. ^i^Blood glucose level was known in 1,286 (94%) episodes admitted to ICU and 1,240 (93%) admitted to other wards. ^j^CSF leukocyte count was known in 1,293 (94%) episodes admitted to ICU and 1,294 (97%) admitted to other wards. ^k^CSF:blood glucose ratio was known in 1,226 (90%) episodes admitted to ICU and 1,198 (89%) admitted to other wards. ^l^CSF protein level was known in 1,270 (93%) episodes admitted to ICU and 1,270 (95%) admitted to other wards.

**Additional file 12: Table S3** Baseline and clinical characteristics of episodes transferred to ICU later during admission (N = 204)

| **Characteristic** | **Data** | **Characteristic** | | **Data** |
| --- | --- | --- | --- | --- |
| Median age (IQR), years^a^ | 66 (56-73) | **Causative pathogen** |  | |
| Female sex | 99/204 (49%) | *S. pneumoniae* | | 135/204 (66%) |
| Symptoms <24 h | 80/195 (41%) | *N. meningitidis* | | 11/204 (5%) |
| Recurrent meningitis | 11/204 (5%) | *H. influenzae* | | 4/204 (2%) |
| Extrameningeal infection | 81/203 (40%) | *L. monocytogenes* | | 31/204 (15%) |
| Otitis or sinusitis | 52/195 (27%) | Positive blood culture | | 137/170 (81%) |
| Endocarditis | 10/195 (5%) | **Radiological examination on admission** | | |
| Pneumonia | 27/197 (14%) | Abnormal CT/MRI brain scan | | 89/204 (44%) |
| Immunocompromised state | 67/204 (33%) | Generalized edema | | 13/175 (7%) |
| **Symptoms on admission** |  | Hydrocephalus | | 12/176 (7%) |
| Headache | 121/170 (71%) | Hypodensity (recent) | | 14/169 (8%) |
| Nausea | 92/166 (55%) | Mastoid opacification | | 26/168 (15%) |
| Neck stiffness | 125/181 (69%) | Sinus opacification | | 34/167 (20%) |
| Rash | 10/183 (5%) | **Clinical course** | |  |
| Median temperature (°C)^b^ | 38.5 (37.3-39.3) | Pneumonia | | 55/182 (30%) |
| Fever (>38°C) | 127/199 (64%) | Circulatory shock | | 42/177 (24%) |
| Heart rate (beats/minute)^c^ | 99 (84-111) | Respiratory failure | | 120/191 (63%) |
| Systolic blood pressure (mmHg)^d^ | 142 (125-162) | Mechanical ventilation | | 129/190 (68%) |
| Glasgow Coma Scale Score^f^ | 11 (10-14) | Seizures | | 59/192 (31%) |
| Altered mental status (GCS <14) | 143/204 (70%) | Cerebrovascular accident | | 32/185 (17%) |
| Coma (GCS <8) | 23/204 (11%) | Sinus thrombosis | | 4/178 (2%) |
| Seizures | 13/191 (7%) | **Outcome** | |  |
| Cranial nerve palsy | 14/174 (8%) | Total duration of hospital stay (days)^p^ | | 23 (15-38) |
| Mono-, hemiparesis or aphasia | 53/181 (29%) | Days to death | | 11 (5-16) |
| **Blood results** |  | Glasgow Outcome Score | |  |
| C-reactive protein (mg/L)^g^ | 219 (94-332) | 1 (death) | | 62/204 (30%) |
| Leukocytes (x10^9/L)^h^ | 15 (11-21) | 2 (vegetative state) | | 1/204 (0%) |
| Thrombocytes (x10^9/L)^i^ | 195 (145-253) | 3 (severe disability) | | 16/204 (8%) |
| **CSF chemistry** |  | 4 (moderate disability) | | 56/204 (27%) |
| Opening pressure (cm H2O)^k^ | 34 (27-50) | 5 (mild or no disability) | | 69/204 (34%) |
| Protein (g/L)^l^ | 4.2 (2.7-6.6) | **Neurological sequelae** | |  |
| CSF:blood glucose ratio^n^ | 0.05 (0.01-0.21) | Cognitive impairment | | 37/109 (34%) |
| White cell count (per mm^3^)^o^ | 1,135 (206-3,924) | Cranial nerve palsy | | 13/114 (11%) |
| <100 per mm^3^ | 36/198 (18%) | Mono-, hemiparesis or aphasia | | 23/121 (19%) |
| 100-999 per mm3 | 59/198 (30%) | Hearing impairment | | 35/135 (26%) |
| >999 per mm3 | 103/198 (52%) |  | |  |
| Data presented as n/N (%) or median (IQR). CSF = cerebrospinal fluid. LP = lumbar puncture. ICU = intensive care unit. | | | | |
| ^a^ Age was known in 204 (100%) episodes. ^b^ Temperature was known in 199 (98%) episodes. ^c^ Heart rate was known in 195 (96%)  episodes. ^d^ Systolic blood pressure was known in 197 (97%) episodes. ^f^ Glasgow Coma Scale Score was known in 204 (100%)  episodes. ^g^ C-reactive protein level was known in 197 (97%) episodes. ^h^ Blood leukocyte count was known in 199 (98%) episodes.  ^i^ Blood thrombocyte count was known in 186 (91%) episodes. ^k^ Opening pressure in CSF was known in 73 (36%) episodes. ^l^ CSF  protein level was known in 192 (94%) episodes. ^n^ CSF:blood glucose ratio was known in 174 (85%) episodes. ^o^ CSF white cell  count was known in 198 (97%) episodes. ^p^ Duration of stay was known in 135 (66%) episodes (episodes that died were not included). ^q^ Days to death was known in 58 (94%) episodes. | | | | |
